# Risk Factors and Structural Impact of Macular Edema in Preterm Infants: An OCT-Based Cohort Study

**DOI:** 10.1167/iovs.67.5.12

**Published:** 2026-05-06

**Authors:** Weiming Yang, Xiaohong Zhou, Yian Li, Xiaojing Cai, Chenhao Yang

**Affiliations:** 1 Department of Ophthalmology, Children's Hospital of Fudan University, National Children's Medical Center, Shanghai, China

**Keywords:** preterm infants, macular edema, optical coherence tomography (OCT), foveal development, risk factors

## Abstract

**Purpose:**

To investigate risk factors for macular edema (ME) and its impact on foveal development in preterm infants using handheld optical coherence tomography (OCT) and clinical data.

**Methods:**

This retrospective cohort included 106 preterm infants who underwent routine retinopathy of prematurity screening with RetCam 3 and OCT. Clinical variables were collected from records. ME was classified by OCT morphology. Quantitative foveal parameters—central foveal thickness (CFT), foveal inner retinal thickness (FIRT), foveal outer retinal thickness (FORT), mean parafoveal thickness (MPFT), mean parafoveal inner retinal thickness (MPIRT), foveal depth (FD), and foveal angle (FA)—were measured. Logistic regression identified ME risk factors, and linear and mixed-effects models assessed structural impact.

**Results:**

Of 106 infants, 27 developed ME (25.5%), and 79 served as controls (74.5%). Infants with resolved ME showed persistent abnormalities, including increased CFT (β = 43.40, *P* < 0.001), FORT (β = 27.67, *P* < 0.001), MPFT (β = 109.91, *P* < 0.001), and FA (β = 20.82, *P* < 0.001). MPIRT was also increased (β = 58.70, *P* = 0.002), whereas FD and FIRT did not differ significantly. Independent risk factors for ME included higher postmenstrual age at OCT, longer phototherapy, respiratory support, and moderate-to-high nutritional risk. Birth weight, gestational age, and Apgar scores were not significant predictors.

**Conclusions:**

ME in preterm infants is associated with persistent foveal abnormalities despite apparent resolution on OCT. Its development is more closely related to postnatal systemic factors—including nutrition, respiratory support, and phototherapy—than to perinatal indices, supporting risk-based OCT monitoring and longer follow-up.

Retinopathy of prematurity (ROP) remains a major cause of visual morbidity in children worldwide, particularly as advances in neonatal care have increased survival among extremely preterm infants.^1^^,^^2^ Beyond the vascular abnormalities that define ROP, structural changes in the macula have been increasingly recognized as important contributors to long-term visual outcomes. Handheld spectral-domain optical coherence tomography (OCT) has enabled in vivo visualization of these abnormalities at the bedside, revealing a high prevalence of macular alterations in preterm infants (including those without clinically apparent ROP), findings rarely observed in term-born infants.^3^^,^^4^

Among these abnormalities, cystoid macular edema (CME) is reported in 30% to 60% of preterm infants between 30 and 43 weeks postmenstrual age (PMA). It is characterized by intraretinal cystic spaces and increased foveal thickness, yet its mechanisms and clinical relevance remain uncertain.^4^^,^^5^ Although some infants demonstrate spontaneous resolution, it is unclear whether CME leaves lasting sequelae on foveal maturation and visual function.^6^^,^^7^ Proposed risk factors include lower gestational age, lower birth weight, oxygen therapy, and perinatal inflammation, but their independent contributions remain poorly defined.^5^

Recent cohort studies have further shown that foveal differentiation, including inner retinal layer displacement and formation of a deep foveal pit, is often incomplete in extremely preterm infants and may persist through term-equivalent age or beyond.^2^^,^^8^ Longitudinal studies also suggest that the presence of CME is associated with delayed photoreceptor development, increased hyperopia, and poorer neurodevelopmental outcomes.^9^^,^^10^ However, most prior investigations have been limited by small sample sizes, single-center designs, short follow-up, and the absence of standardized OCT grading criteria, restricting comparability across studies. Moreover, few have systematically evaluated systemic and perinatal risk factors alongside structural OCT outcomes and long-term refractive or functional endpoints.^1^

In this study, we aimed to clarify the clinical risk factors for macular edema (ME) in preterm infants and to determine its impact on foveal development using detailed OCT imaging and standardized perinatal data collection. Unlike previous reports, our work integrated quantitative OCT measurements with comprehensive medical histories, allowing a more robust evaluation of both systemic and ocular predictors of ME. Our retrospective cohort included preterm infants who underwent regular handheld OCT screening during ROP surveillance, with longitudinal assessment of foveal structure and maturation. By correlating OCT features with clinical variables, we hoped to identify early markers of impaired foveal development and provide new evidence to guide screening and intervention strategies for at-risk preterm infants.

## Methods

### Ethics Approval

This study was approved by the Ethics Committee of Children's Hospital of Fudan University (Approval No. 267 [2019]) and conducted in accordance with the tenets of the Declaration of Helsinki. Written informed consent was obtained from the parents or legal guardians of all participants.

### Study Design and Participants

This retrospective cohort study included preterm infants (<37 weeks gestation) who underwent ROP screening at Children's Hospital of Fudan University from January 2022 to December 2023. Inclusion criteria included (1) gestational age < 37 weeks; (2) high-quality macular images from both RetCam 3 fundus photography (Clarity Medical Systems, Pleasanton, CA, USA) and handheld spectral-domain OCT (Envisu C2300; Leica Microsystems, Wetzlar, Germany); and (3) availability of complete demographic and perinatal clinical data. All included cases of ROP, when present, were mild (zones II and III, stages 1 and 2, plus-negative) according to the International Classification of Retinopathy of Prematurity (ICROP), and they had regressed spontaneously without treatment. Exclusion criteria included (1) congenital ocular anomalies or genetic/metabolic diseases affecting macular morphology; (2) history of retinal interventions (e.g., laser, anti-vascular endothelial growth factor [VEGF]); and (3) poor OCT image quality or incomplete medical records.

### Clinical and Imaging Data Collection

Demographic and perinatal data—including gestational age, PMA at OCT, birth weight, sex, delivery mode, Apgar scores, hospitalization duration, respiratory support, ROP status, infection history, nutrition risk, Score for Neonatal Acute Physiology-II (SNAP-II) score, phototherapy history, and details at onset of ME—were extracted from electronic medical records.

### OCT Imaging and Analysis

OCT and fundus images were obtained at the bedside during ROP screening by experienced ophthalmologists. As part of routine ROP screening, each infant underwent serial handheld OCT imaging using line-scan mode. During each imaging session, three to six consecutive B-scans were typically acquired at the same macular location to ensure sufficient signal and minimize motion artifacts. For quantitative analysis, one representative OCT image per eye was selected from each imaging session based on image quality, defined by the highest signal-to-noise ratio and minimal motion artifact. When multiple images from the same session met these criteria, the first qualifying high-quality image was selected for analysis. Infants with ME were followed longitudinally until resolution or discharge. All OCT images were independently evaluated by two trained graders blinded to clinical data, with discrepancies adjudicated by a senior ophthalmologist.

### OCT Image Analysis and Classification

All OCT images were independently evaluated by two ophthalmologists with standardized training, blinded to clinical data. ME was identified and classified based on time of onset and morphological characteristics. Foveal developmental parameters assessed included the following:

1. Foveal depth (FD), distance from the lowest point of the foveal pit to the nasal and temporal peaks
2. Central foveal thickness (CFT), from the internal limiting membrane (ILM) to the retinal pigment epithelium (RPE)
3. Foveal inner retinal thickness (FIRT), from the ILM to the inner nuclear layer (INL)
4. Foveal outer retinal thickness (FORT), from the INL boundary to the RPE (FORT = CFT – FIRT).
5. Mean parafoveal thickness (MPFT), mean thickness at 1000 µm nasal and temporal to the fovea
6. Mean parafoveal inner retinal thickness (MPIRT), mean parafoveal inner retinal layer thickness at nasal and temporal points
7. Foveal angle (FA), angle formed at the foveal pit between nasal and temporal walls

**Figure 1.**
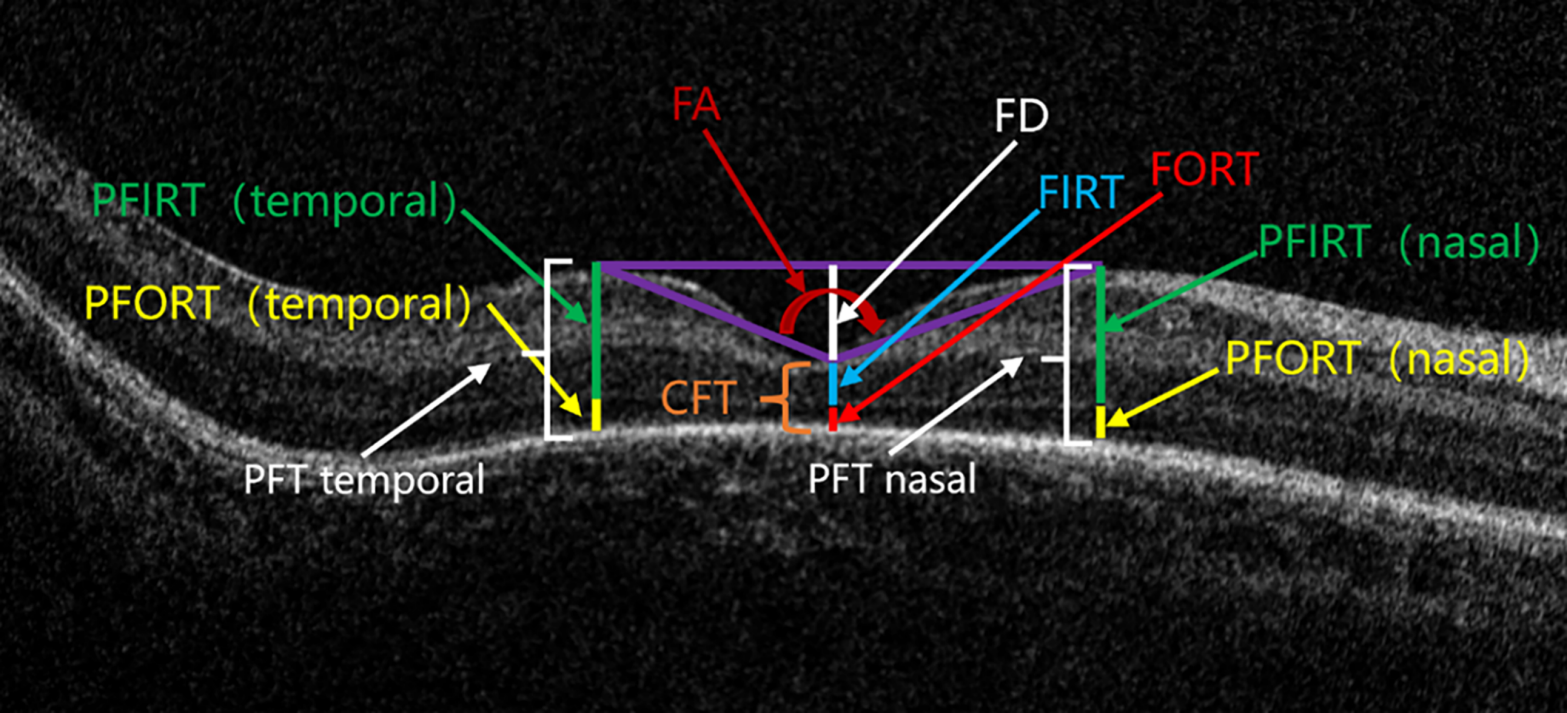
**Quantitative parameters of foveal and parafoveal morphology measured on spectral-domain OCT (SD-OCT).** Representative SD-OCT image of the central macula showing all quantitative measurements. *Colored arrows* indicate the location of each parameter as follows: FD (*white*), CFT (*orange*), FIRT (*light blue*), FORT (*red*), MPFT (*white brackets* for nasal and temporal locations), MPIRT (*green*, nasal and temporal), mean parafoveal outer retinal thickness (MPFORT; *yellow*, nasal and temporal), FA (*red angle* at the foveal pit vertex). FD (*white*) is the vertical distance from the lowest point of the foveal pit to a line drawn between the nasal and temporal parafoveal peaks. CFT (*orange*) is the distance from the ILM to the RPE at the foveal center. FIRT (*light blue*) is the thickness from the ILM to the boundary of the INL at the foveal center. FORT (*red*) is the thickness from the INL boundary to the RPE at the foveal center, calculated as CFT minus FIRT. MPFT (*white*) is the average retinal thickness measured at 1000 µm nasal and temporal to the foveal center (parafoveal thickness nasal and parafoveal thickness temporal). MPIRT (*green*) is the mean thickness from the ILM to the INL boundary at 1000 µm nasal and temporal to the foveal center (PFIRT nasal and PFIRT temporal). MPFORT (*yellow*) is the mean thickness from the INL boundary to the RPE at 1000 µm nasal and temporal to the foveal center (PFORT nasal and PFORT temporal). FA (*red*) is the angle at the vertex of the foveal pit formed by lines connecting the lowest point of the pit to the nasal and temporal parafoveal peaks.

All parameters were measured independently twice by each grader, and means were used for analysis. In case of substantial discrepancy, a senior ophthalmologist determined the final measurement (Fig. 1).

### Statistical Analysis

All analyses were performed using R 4.4.3 (R Foundation for Statistical Computing, Vienna, Austria). Baseline characteristics were summarized as mean ± standard deviation (SD) or median (interquartile range) for continuous variables and as counts (percentages) for categorical variables. Normality was assessed using the Shapiro–Wilk test. Between-group comparisons for continuous variables were performed using Student's *t*-test when the normality assumption was met or the Mann–Whitney *U* test otherwise. For categorical variables, the χ^2^ test was used when expected cell frequencies were ≥5, and Fisher's exact test was applied for smaller cell counts. Univariate logistic regression was first applied to screen potential risk factors for ME, followed by multivariable logistic regression to identify independent predictors while adjusting for confounders. Odds ratios (ORs) with 95% confidence intervals (CIs) were reported. To evaluate the effect of ME on foveal structural parameters, both single-eye linear regression and mixed-effects models were applied. For single-eye regression, only left-eye data were analyzed to avoid inter-eye correlation. Mixed-effects models incorporated data from both eyes while accounting for within-subject correlation. For both approaches, two sets of models were tested: (1) fully adjusted for sex, PMA at OCT, and birth weight; and (2) adjusted for PMA only. Regression coefficients (β) with 95% CIs were reported. All statistical tests were two sided, and *P* < 0.05 was considered statistically significant.

### Quality Control

Standardized protocols were applied throughout. Measurement consistency was confirmed by intraclass correlation coefficients (ICCs) > 0.9. Data collection and analysis were independently performed to minimize bias.

## Results

This study included 106 preterm infants. Of these, 79 infants (74.5%) showed no evidence of ME during follow-up and were classified as the control group, whereas 27 infants (25.5%) developed ME on at least one OCT scan. Of the 27 infants with ME, 23 had bilateral involvement (85.2%), whereas four had unilateral edema (14.8%). Among the edema group, nine infants completed longitudinal follow-up with confirmed resolution of edema.

At the time of OCT imaging, the mean PMA was 45.46 ± 5.8 weeks in the resolved ME group and 37.83 ± 3.2 weeks in the control group. The mean gestational ages at birth were 34.94 ± 2.7 weeks and 32.31 ± 3.4 weeks, respectively. The mean birth weight was 1861.7 ± 541.7*g* for the ME group and was 1764.2 ± 603.8*g* for controls. Male sex accounted for 55.6% of the ME group and 64.6% of the control group (Table 1).

**Table 1.** Baseline Characteristics of Preterm Infants With a History of Resolved ME and Those Without ME

Characteristic	Control (*n* = 79)	ME (*n* = 9)
PMA at imaging (wk), mean ± SD	37.83 ± 3.2	45.46 ± 5.8
Gestational age (wk)	32.31 ± 3.4	34.94 ± 2.7
Birth weight (g), mean ± SD	1764.2 ± 603.8	1861.7 ± 541.7
Male sex, *n* (%)	51 (64.6)	15 (55.6)

**Figure 2.**
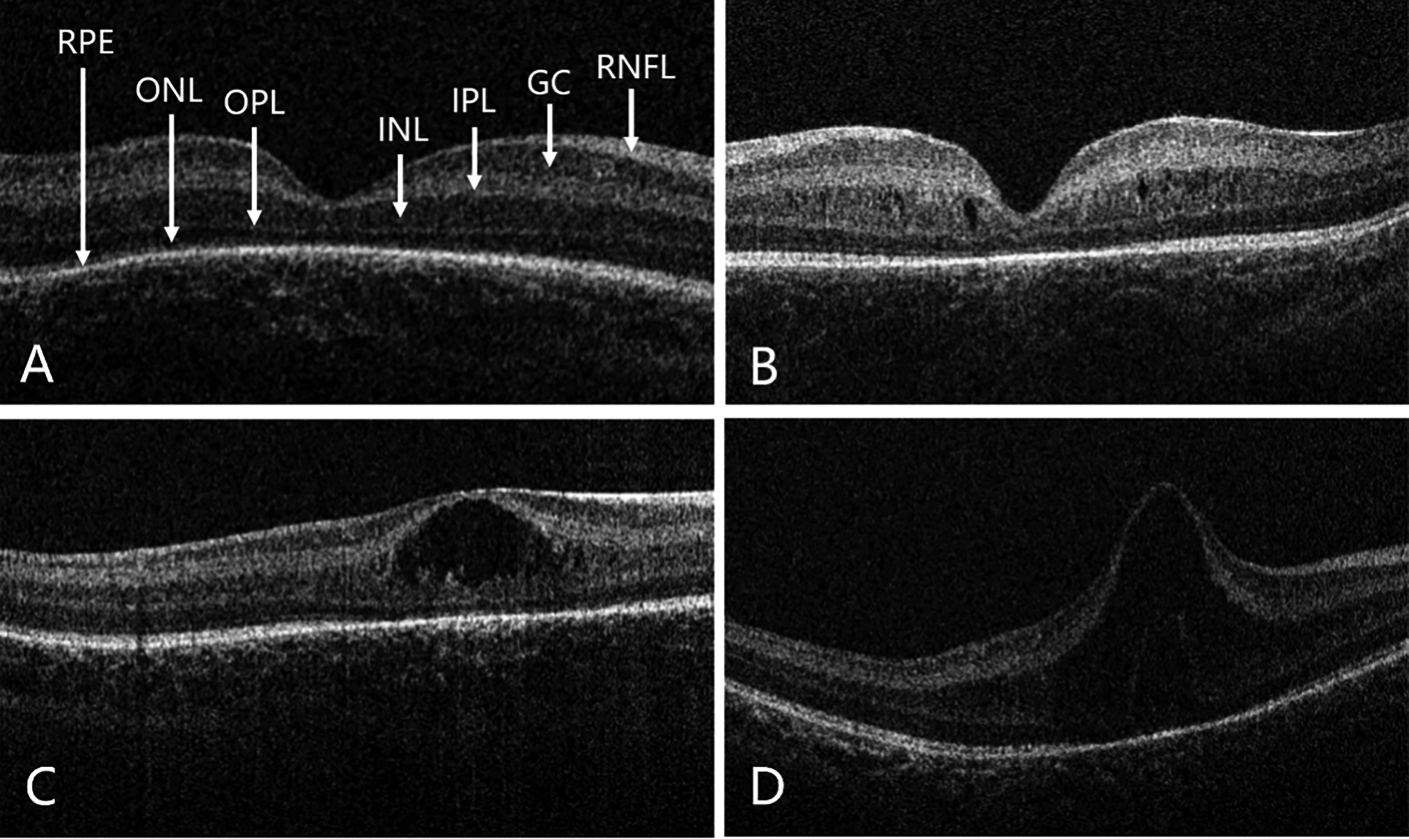
**Representative OCT images of foveal morphology in preterm infants.** (**A**) Normal but incompletely developed foveal structure in a preterm infant, with retinal layers labeled: RPE, outer nuclear layer (ONL), outer plexiform layer (OPL), INL, inner plexiform layer (IPL), ganglion cell layer (GC), and retinal nerve fiber layer (RNFL). (**B**) Mild ME, characterized by small cystoid hyporeflective spaces within the INL while preserving overall foveal contour. (**C**) Moderate ME, showing increased INL cystic spaces with mild foveal elevation and flattening of the foveal pit. (**D**) Severe ME, with marked foveal elevation, large confluent cystoid cavities within the INL, and severe disruption of the normal foveal architecture.

In the edema group, based on initial OCT morphology, nine infants presented with mild edema (33.3%), characterized by small intraretinal cystoid spaces within the INL with preserved foveal contour. Moderate edema was observed in 11 infants (40.7%), defined by increased cystic spaces with mild foveal elevation or flattening. Severe edema was observed in seven infants (25.9%), with marked foveal elevation, extensive cystic changes, and significant disruption of foveal architecture (Table 1; Fig. 2).

### Effect of ME on Foveal Structural Development

To evaluate the long-term impact of ME on foveal development, we compared nine infants with resolved edema (case group) to 79 infants without edema (control group), for a total of 88 subjects (176 eyes). To ensure model robustness, we performed both single-eye linear regression (using left-eye data only) and mixed-effects models incorporating both eyes. Two adjustment strategies were applied: (1) full adjustment for sex, PMA at imaging, and birth weight; and (2) minimal adjustment for PMA only. Variance inflation factors (VIFs; all < 2) and heteroskedasticity-consistent 3 (HC3) robust standard errors confirmed the absence of multicollinearity and heteroscedasticity (Table 2).

**Table 2.** VIFs for Covariates Included in Regression Models

Variable	VIF
Status^*^	1.46
Sex	1.02
PMA at imaging	1.82
Birth weight	2.33
Gestational age	2.86

VIFs were calculated to assess multicollinearity among covariates in the regression analyses. All VIF values were well below the conventional threshold of 5, indicating no significant collinearity.

* Status indicates a history of resolved ME (0 = control, 1 = case).

The primary analysis was based on a mixed-effects model including both eyes, adjusted only for PMA. Infants with a history of resolved ME demonstrated significantly greater CFT (β = 43.40, *P* < 0.001), FORT (β = 27.67, *P* < 0.001), MPFT (β = 109.91, *P* < 0.001), and FA (β = 20.82, *P* < 0.001) compared with controls. MPIRT was also significantly increased (β = 58.70, *P* = 0.002), whereas FD and FIRT did not differ significantly between groups (all *P* > 0.05) (Table 3).

**Table 3.** Association Between Prior ME and Foveal Structural Parameters Across Four Regression Models

Parameter	LM_full, β (*P*)	LM_pma, β (*P*)	LMM_full, β (*P*)	LMM_pma, β (*P*)
FD	−24.31 (0.149)	−24.55 (0.101)	−22.61 (0.107)	−22.03 (0.114)
CFT	44.52 (**0.003**)	48.31 (**0.002**)	40.62 (**<0.001**)	43.40 (**<0.001**)
FIRT	14.71 (0.1)	18.16 (0.085)	12.26 (0.249)	15.73 (0.145)
FORT	29.81 (**0.004**)	30.15 (**0.004**)	28.35 (**<0.001**)	27.67 (**<0.001**)
MPFT	120.87 (**0.044**)	121.30 (**0.049**)	110.47 (**<0.001**)	109.91 (**<0.001**)
MPIRT	70.01 (0.07)	70.88 (0.085)	58.35 (**0.002**)	58.70 (0.002)
FA	18.96 (**<0.001**)	19.99 (**<0.001**)	20.32 (**<0.001**)	20.82 (**<0.001**)

Bold *P* values indicate statistical significance (*P* < 0.05). β, regression coefficient (case vs. control group, adjusted as noted); LM_full, linear regression (sex, PMA, body weight adjusted); LM_pma, linear regression (PMA only); LMM_full, mixed-effects (sex, PMA, body weight adjusted); LMM_pma, mixed-effects (PMA only).

Across all four models—full-adjusted single-eye linear regression (LM_full), PMA-adjusted single-eye linear regression (LM_pma), full-adjusted linear mixed-effects model (LMM_full), and PMA-adjusted linear mixed-effects model (LMM_pma)—findings were highly consistent. Compared with controls, infants with a history of resolved edema exhibited persistent increases in CFT, FORT, MPFT, and FA, with all differences reaching statistical significance (all *P* < 0.01 for CFT, FORT, and FA; *P* < 0.05 for MPFT in three models). MPIRT was significantly greater in both mixed-effects models and approached significance in single-eye models. No significant differences were observed for FD or FIRT in any model (Table 3).

### Risk Factor Analysis for ME

Clinical and demographic characteristics were compared between 27 infants with ME and 79 controls. Significant differences were observed between the two groups in several clinical parameters. Infants with ME had a higher PMA at OCT imaging (41.26 ± 3.27 weeks vs. 37.83 ± 3.25 weeks; *P* < 0.001), longer duration of anti-infective therapy (22.56 ± 16.31 days vs. 14.70 ± 11.71 days; *P* = 0.029), higher SNAP-II scores (3.63 ± 4.29 vs. 0.82 ± 2.38; *P* < 0.001), longer phototherapy duration (6.77 ± 4.75 days vs. 3.73 ± 3.94 days; *P* = 0.002), greater need for respiratory support (88.9% vs. 48.1%; *P* < 0.001), higher rates of moderate-to-high nutritional risk (48.1% vs. 10.1%; *P* < 0.001), and a higher incidence of ROP (37.0% vs. 16.5%; *P* = 0.025). In contrast, no significant differences were found between infants with and without ME in birth weight (1764.24 ± 603.82*g* vs. 1614.07 ± 557.03*g*; *P* = 0.319), gestational age (32.31 ± 3.42 weeks vs. 32.10 ± 4.01 weeks; *P* = 0.980), gravida (2.29 ± 1.54 vs. 2.00 ± 1.52; *P* = 0.191), parity (1.49 ± 0.57 vs. 1.30 ± 0.47; *P* = 0.127), hospital length of stay (43.73 ± 60.86 days vs. 43.33 ± 31.23 days; *P* = 0.744), sex distribution (male: 64.6% vs. 55.6%; *P* = 0.340), mode of delivery (cesarean section: 67.1% vs. 55.6%; *P* = 0.281), and Apgar scores at 1 minute (11.4% vs. 25.9%; *P* = 0.115) and 5 minutes (6.3% vs. 14.8%; *P* = 0.228) (Table 4).

**Table 4.** Baseline Characteristics of Infants With and Without ME

Variable	Control Group (*n* = 79)	ME Group (*n* = 27)	*P*
Birth weight (g), mean ± SD	1764.24 ± 603.82	1614.07 ± 557.03	0.319
Gestational age (wk), mean ± SD	32.31 ± 3.42	32.10 ± 4.01	0.980
PMA at imaging (wk), mean ± SD	37.83 ± 3.25	41.26 ± 3.27	**<0.001**
Gravida, mean ± SD	2.29 ± 1.54	2.00 ± 1.52	0.191
Parity, mean ± SD	1.49 ± 0.57	1.30 ± 0.47	0.127
Hospital length of stay (d), mean ± SD	43.73 ± 60.86	43.33 ± 31.23	0.744
Infection duration (d), mean ± SD	14.70 ± 11.71	22.56 ± 16.31	**0.029**
SNAP-II score, mean ± SD	0.82 ± 2.38	3.63 ± 4.29	**<0.001**
Phototherapy duration (d), mean ± SD	3.73 ± 3.94	6.77 ± 4.75	**0.002**
Male, *n* (%)	51 (64.6)	15 (55.6)	0.340
Respiratory support required, *n* (%)	38 (48.1)	24 (88.9)	**<0.001**
ROP, *n* (%)	13 (16.5)	10 (37.0)	**0.025**
Cesarean delivery, *n* (%)	53 (67.1)	15 (55.6)	0.281
Apgar score < 7 at 1 min, *n* (%)	9 (11.4)	7 (25.9)	0.115
Apgar score < 7 at 5 min, *n* (%)	5 (6.3)	4 (14.8)	0.228
Moderate/high nutrition risk, *n* (%)	8 (10.1)	13 (48.1)	**<0.001**

*P* values are from Student's *t*-tests or χ^2^ tests as appropriate; statistically significant *P* values (*P* < 0.05) are shown in bold. The control group included infants without ME during follow-up. The ME group included infants diagnosed with ME on at least one OCT scan. Moderate/high nutrition risk was defined according to standardized screening criteria.

In univariate logistic regression analysis, Bonferroni correction was applied to account for multiple comparisons (significance threshold: *P* < 0.0031; i.e., 0.05/16). After adjustment, five variables remained significantly associated with ME: higher PMA at imaging (OR = 1.35; 95% CI, 1.16–1.57; *P* < 0.001), higher SNAP-II score (OR = 1.28; 95% CI, 1.12–1.46; *P* < 0.001), longer phototherapy duration (OR = 1.18; 95% CI, 1.06–1.31; *P* = 0.003), need for respiratory support (OR = 8.63; 95% CI, 2.40–31.01; *P* < 0.001), and moderate-to-high nutritional risk (OR = 8.24; 95% CI, 2.88–23.57; *P* < 0.001). Variables including infection duration, presence of ROP, birth weight, gestational age, gravida, parity, hospital length of stay, sex, mode of delivery, and Apgar scores did not reach statistical significance after correction (all adjusted *P* ≥ 0.0031) (Table 5).

**Table 5.** Univariate Logistic Regression Analysis of Risk Factors for ME in Preterm Infants

Variable	OR	95% CI	*P*
Birth weight (per g)	1.00	1.00–1.00	0.298
Gestational age (per wk)	0.99	0.87–1.12	0.819
PMA (per wk)	1.35	1.16–1.57	**<0.001**
Gravida	0.87	0.63–1.20	0.395
Parity	0.49	0.20–1.18	0.113
Hospital length of stay (per d)	1.00	0.99–1.01	0.974
Infection duration (per d)	1.04	1.01–1.08	0.012
SNAP-II score (per point)	1.28	1.12–1.46	**<0.001**
Phototherapy duration (per d)	1.18	1.06–1.31	**0.003**
Male sex	1.54	0.63–3.75	0.341
Respiratory support (yes vs. no)	8.63	2.40–31.01	**<0.001**
ROP (yes vs. no)	2.99	1.12–7.97	0.029
Cesarean delivery (yes vs. no)	0.61	0.25–1.50	0.283
Apgar score < 7 at 1 min (yes vs. no)	2.72	0.90–8.22	0.076
Apgar score < 7 at 5 min (yes vs. no)	2.57	0.64–10.39	0.184
Moderate/high nutrition risk	8.24	2.88–23.57	**<0.001**

Odds ratios represent the change in odds of developing ME per unit increase in continuous variables or for the presence versus absence of categorical variables. *P* values are based on Wald tests. When Bonferroni correction was applied, *P* < 0.0031 (0.05/16) was considered statistically significant; statistically significant *P* values are shown in bold.

Variables with *P* < 0.05 in univariate analysis were entered into the multivariable model. Four independent risk factors were identified: higher PMA at OCT (OR = 1.62; 95% CI, 1.26–2.08; *P* < 0.001), longer phototherapy duration (OR = 1.21; 95% CI, 1.03–1.42; *P* = 0.019), moderate-to-high nutritional risk (OR = 7.69; 95% CI, 1.65–35.79; *P* = 0.009), and need for respiratory support (OR = 6.62; 95% CI, 1.18–37.02; *P* = 0.032). SNAP-II score did not remain significant after adjustment (*P* = 0.471) (Table 6).

**Table 6.** Multivariable Logistic Regression Analysis of Independent Risk Factors for ME in Preterm Infants

Variable	OR	95% CI	*P*
PMA (per wk)	1.62	1.26–2.08	<0.001
SNAP-II score (per point)	1.08	0.87–1.35	0.471
Phototherapy duration (per d)	1.21	1.03–1.42	**0.019**
Respiratory support (yes vs. no)	6.62	1.18–37.02	**0.032**
Moderate/high nutrition risk	7.69	1.65–35.79	**0.009**

Odds ratios represent the change in odds of developing ME per unit increase in continuous variables or for the presence versus absence of categorical variables. *P* values are based on Wald tests. Statistically significant *P* values are shown in bold.

## Discussion

In this single-center retrospective cohort, ME in preterm infants was independently associated with several postnatal care–related factors: higher PMA at imaging, longer phototherapy duration, moderate-to-high nutritional risk, and the need for respiratory support. Even when ME appeared to have resolved on imaging, infants with a history of ME continued to show structural differences in the fovea. These included increased CFT, FORT, greater MPFT, and a wider FA. FD and FIRT did not differ significantly between groups. MPIRT was significantly greater only in mixed-effects models. These patterns were consistent across both single-eye and mixed-effects analyses, under both full and minimal adjustment strategies. All VIFs were <2, robust HC3 standard errors were applied, and intergrader agreement was excellent (ICC > 0.9), supporting the robustness of the findings.

Previous studies have reported that the prevalence of ME in preterm infants ranges from approximately 30% to 60%, with variability largely attributable to differences in cohort composition, imaging timing, and grading criteria.^4^^,^^10^^,^^11^ In our cohort, the prevalence was 25.5% (27/106 infants), which is slightly below the lower limit of this reported range. This lower incidence may reflect our inclusion of a wider spectrum of PMAs at imaging, stricter image quality thresholds, and potentially more conservative diagnostic criteria.

In addition, the baseline characteristics of our cohort differed from several prior studies, as our infants were generally older and had higher birth weights at the time of OCT imaging. Earlier investigations^7^^,^^8^^,^^10^ primarily imaged younger and smaller infants (mean PMA = 32–38 weeks) and reported a higher prevalence of ME with more prominent cystoid changes. In contrast, our imaging occurred closer to term-equivalent age (mean PMA ≈ 45 weeks), when retinal lamination is more advanced and the inner layers have begun to thin. This later imaging window may explain the lower edema prevalence (25.5%) and the predominance of mild parafoveal cystoid changes with relatively preserved foveal contour in our cohort. These differences highlight how developmental stage and imaging timing influence both the incidence and morphology of ME. Rather than contradicting earlier work, our results complement existing data by representing a later stage in the developmental spectrum of ME, when edema is resolving but persistent outer retinal thickening and foveal flattening remain visible.

Several studies have further demonstrated that ME in preterm infants is significantly associated with the stage of ROP, particularly stages 2 and 3 disease, and with ocular features such as zone I involvement, whereas consistent associations with birth weight, gestational age, or systemic conditions have not been observed.^4^^,^^6^^,^^10^ In our cohort, ROP was present in 10 of 27 infants with ME (37.0%) and in 13 of 79 controls (16.5%), showing a nominal difference between the two groups (*P* = 0.025). Univariate logistic regression analysis further suggested that ROP-positive infants had approximately threefold higher odds of developing ME compared with ROP-negative infants (OR = 2.986; 95% CI, 1.119–7.971; unadjusted *P* = 0.029). However, after Bonferroni correction for multiple comparisons (adjusted significance threshold *P* < 0.0031), the association was no longer statistically significant, and ROP status was not retained in the multivariable model.

All ROP cases in this study were mild (zones II and III, stages 1 and 2, plus-negative) and regressed spontaneously without treatment. The lack of statistical significance likely reflects the limited disease severity and absence of therapeutic interventions, such as laser photocoagulation or intravitreal anti-VEGF injections, which have been shown in previous studies to influence retinal vascular permeability and increase macular fluid accumulation.^4^^,^^10^ Nonetheless, the trend observed in our data suggests that even mild vascular immaturity associated with ROP could transiently compromise the blood–retina barrier or modulate intraocular VEGF signaling, predisposing some infants to ME. Larger multicenter studies including a broader range of ROP severities and treatment exposures will be required to confirm whether ROP represents an independent mechanistic contributor or a secondary indicator of systemic vulnerability in foveal development.

In our study, baseline “at-birth” indicators such as gestational age and birth weight were not primary determinants of ME. Instead, variables reflecting postnatal course and intensity of care, including higher PMA at imaging, requirement for respiratory support, moderate-to-high nutritional risk, and longer phototherapy duration, showed stronger associations with ME occurrence. These findings are in line with recent evidence that postnatal exposures influence foveal differentiation and that ME is more likely to develop and be detected closer to term-equivalent age.^2^^,^^5^^,^^8^ Our observation regarding nutritional risk is also consistent with prior studies demonstrating that adequate early provision of energy, protein, and key lipids such as docosahexaenoic acid and arachidonic acid supports optimal retinal and neurovascular development in preterm infants.^12^^,^^13^

In our cohort, the macular architecture following ME was characterized by persistent outer retina–dominant remodeling. Specifically, infants with prior ME exhibited increases in CFT, FORT, MPFT, and FA, whereas FD and FIRT remained comparable to controls. MPIRT showed an increasing trend and reached statistical significance only in mixed-effects models.

Beyond structural alterations, the temporal and morphological characteristics of ME in our cohort warrant further discussion. Previous studies in preterm infants suggest that ME may occur during distinct stages of postmenstrual development, with differences in its morphological appearance depending on the timing of onset. Early-onset edema (typically ≤33 weeks PMA) has been characterized by prominent foveal bulging and elongated cystoid spaces, whereas late-onset edema (≥36 weeks PMA) more commonly presents as smaller cystoid spaces located parafoveally with a relatively preserved foveal contour, suggesting potentially different underlying mechanisms for these phenotypes.^7^ In our cohort, the mean PMA at imaging among infants with ME was approximately 45 weeks, indicating that the edema observed here predominantly represents the late-onset type. This timing aligns with prior definitions of late-onset edema and may partly explain the relatively localized cystic changes and mild foveal flattening noted in our cohort. Furthermore, morphological variations compared with previous Western studies may reflect differences in imaging timing, neonatal care practices, or subtle population-specific developmental patterns. Prior OCT studies have shown variability in foveal pit formation and retinal layer maturation across populations, suggesting that environmental and developmental factors—rather than ethnicity alone—may influence retinal structural outcomes in preterm infants.^14^ Further multicenter longitudinal studies with harmonized imaging protocols are needed to confirm these observations.

A comparable finding was reported by O'Sullivan et al.,^2^ who found that preterm infants exhibited arrested foveal differentiation with persistent inner retinal layers and shallow foveal pits. This supports the interpretation that transient intraretinal fluid accumulation may disrupt photoreceptor maturation and Henle fiber layer development, resulting in lasting alterations in foveal architecture even after apparent clinical resolution.

The link between respiratory support and ME likely reflects greater illness severity and oxygenation instability, which can weaken the blood–retina barrier and stimulate angiogenic signaling. Similarly, phototherapy—a common treatment for neonatal jaundice—has been shown to cause oxidative stress in preterm infants, with significantly increased thiobarbituric acid–reactive substances after 96 hours of blue-light exposure.^15^ Both factors may contribute to retinal vulnerability and the structural changes seen after ME.

The observed association between higher PMA and ME should not be interpreted causally. PMA serves both as a biological marker of foveal maturation and as a variable influenced by clinical course and imaging schedules. Infants with more severe illness may undergo OCT at later PMAs and more frequently, and ME may be more readily detected near term-equivalent age. Therefore, PMA in this context partly reflects the timing and selection of imaging rather than a direct biological effect. Future studies should implement standardized, sequential handheld OCT protocols at predefined PMA intervals to disentangle true developmental influences from detection bias.

This study supports adding risk-based OCT monitoring to ROP screening. Infants who need respiratory support, have moderate or high nutritional risk, or receive longer phototherapy should be imaged more often and followed closely after discharge. ME that looks “resolved” on imaging can still leave lasting changes, including outer retinal thickening and a flatter foveal pit. These findings highlight the need to track foveal development over time and watch for possible refractive or functional problems. Although we did not test treatments, improving nutrition and reducing avoidable oxygen changes or oxidative stress are reasonable strategies for future studies to confirm.

Strengths of this study include standardized handheld OCT measurements combined with comprehensive perinatal data, independent grading by two reviewers with high agreement (ICC > 0.9), and consistent findings across multiple models with low collinearity (VIF < 2) and HC3 robust standard errors to reduce the impact of model instability and heteroscedasticity. Limitations include the single-center retrospective design, which may have introduced residual confounding and selection bias; the small number of ME cases and limited longitudinal follow-up to complete resolution (*n* = 9); and the fact that some infants had only one OCT scan, limiting assessment of structural trajectories. We also lacked long-term visual function and refractive outcomes, making functional interpretations dependent on prior literature. Finally, the absence of a standardized grading system for foveal maturity and ME severity limits comparisons and meta-analyses across studies.

We recommend conducting multicenter, prospective studies using protocolized, sequential handheld OCT from the early neonatal period through term-equivalent age and into early childhood, with key objectives to: (1) define the time window and determinants of ME onset and resolution; (2) verify whether the “outer retina–predominant thickening with foveal flattening” pattern is an imaging marker of delayed foveal maturation; (3) assess the impact of modifiable exposures—such as nutritional optimization, oxygen/ventilation strategies, and phototherapy practices—on structural development; (4) link structural outcomes with standardized visual function and neurodevelopmental assessments; and (5) promote consensus-based OCT grading and quantitative metrics to enable data pooling and evidence-based decision-making.

## Conclusions

In summary, our study shows that ME in preterm infants is associated with persistent abnormalities in foveal structure, even after edema appears resolved on OCT. Its occurrence is more closely linked to postnatal systemic factors—such as nutritional risk, respiratory support, and phototherapy exposure—than to traditional perinatal indices such as birth weight or gestational age. These findings highlight the importance of comprehensive neonatal management and risk-based OCT monitoring, with extended follow-up to clarify long-term structural and functional consequences. Multicenter, prospective studies are needed to validate these associations and to inform early intervention strategies for this vulnerable population.
